# The Impact of a Positive Body Image Program (Body Image Awareness Seminars) on the Positive and Negative Body Image of Individuals Living With Cancer

**DOI:** 10.1002/cnr2.70161

**Published:** 2025-03-11

**Authors:** Carly A. MaGee, Kimberley L. Gammage

**Affiliations:** ^1^ Faculty of Applied Health Sciences, Department of Kinesiology Brock University Ontario Canada

**Keywords:** body appreciation, body image distress/disturbance, cancer, chronic disease or disability, feasibility, functionality appreciation, group intervention, positive body image, psycho‐oncology, self‐compassion

## Abstract

**Objective:**

Using action research principles to address gaps in existing interventions, and in consultation with members and administrators of Wellspring Canada, the purpose of the current study was to adapt and examine the impact of a novel (concept, content, and inclusivity) positive body image program (Body Image Awareness Seminars; BIAS) on positive and negative body image in individuals living with cancer.

**Methods:**

The project used a single‐group pretest–posttest design with a 6‐week follow‐up. Over the 6‐week program, 37 participants engaged in 90‐min weekly group sessions consisting of researcher‐led psychoeducation, group discussion, and activities grounded in positive body image research. Each seminar had a unique theme designed to promote respect, acceptance, and appreciation for the body. Participants completed the Body Image Scale, Body Appreciation Scale‐2, Functionality Appreciation Scale, Measure of Body Apperception, Appearance Evaluation subscale, and Self‐Compassion Scale anonymously using Qualtrics at the 3 data collection points. Homework assignments included optional readings and writing exercises based on the positive body image tenet taught that week.

**Results:**

Participants reported statistically significant improvements in positive body image (i.e., body appreciation, functionality appreciation), self‐compassion, and negative body image (i.e., body image distress and disturbance, investment in body integrity and appearance evaluation), which were sustained at the 6‐week follow‐up in a subsample of participants (*n* = 11).

**Conclusions:**

This study suggests that the adapted BIAS program can lead to improvements in both positive and negative body image in individuals living with diverse types of cancer. Results support the implementation of BIAS in the standard of care for those living with cancer to continue to affect positive change.

## 1 Background

A relatively significant amount of research has investigated the impact of cancer and its treatment on body image [1, 2, 3]. Body image is a multidimensional construct including thoughts, beliefs, feelings, and behaviors regarding the function and appearance of the body [4]. Body image is affected by sociocultural (e.g., interpersonal relationships, media) and physical (e.g., weight, shape, or body changes such as puberty, menopause, chronic disease) factors [4, 5, 6]. In oncology, body image can vary according to the clinical features of the disease, treatment (with surgical procedures having a greater negative impact on body image than non‐surgical treatments [7, 8]), time since diagnosis (reduced negative body image with longer time since diagnosis [2]), as well as the impact on body functioning (e.g., fatigue, pain [9]), appearance changes (e.g., scarring, weight gain [9]), and age (with younger individuals being more negatively affected [2, 10]). As a result, some individuals living with cancer have reported negative body image outcomes such as feeling embarrassed/ashamed of their bodies, avoidance of people, and appearance dissatisfaction [2, 9, 11].

Negative body image is commonly experienced in those diagnosed and living with cancer. For example, up to 77% of women treated for breast cancer experience some degree of body image concern [12, 13, 14], with little improvement up to 5 years following treatment [15, 16]. Even higher rates of body image concerns (89%) have been reported in individuals living with head and neck cancer following treatment [11]. In addition to the negative cognitive (e.g., dissatisfaction with scars [15, 16, 17]), affective (e.g., loss of femininity/masculinity [16, 17, 18, 19, 20, 21, 22]), and behavioural (e.g., use of make‐up, wigs [16, 17]) dimensions of body image in those living with cancer [5, 23], research has also highlighted detrimental changes to psycho‐emotional balance such as negative interoceptive awareness (e.g., constantly looking for breast lumps) [24].

Given that negative body image in individuals living with cancer is associated with poor health outcomes such as anxiety, depression, sexual and intimacy concerns, and reduced health‐related quality of life [25, 26, 27, 28, 29, 30], there is a need for intervention and improvement. Thus far, body image interventions for individuals living with diverse types of cancer have generally addressed how to reduce body image distress or dissatisfaction [31, 32], in the absence of considering positive body image; one recent review of body image interventions for those living with breast cancer indicated that positive body image was rarely addressed [33].

Positive body image is a multifaceted construct, distinct from negative body image, characterized by: body appreciation (appreciating the body's features, functionality and health, even if it does not meet the societal ideal), body acceptance and love (even in the absence of complete satisfaction), broadly conceptualizing beauty, adaptive appearance investment (appearance‐related self‐care), inner positivity, and filtering information in a body‐protective manner (rejecting negative messages) [34]. Positive body image is positively related to adaptive outcomes such as intuitive eating, self‐compassion, sexual satisfaction, and exercise, and inversely related to perfectionism, diet/weight‐related talk, dieting, and depressive symptom [34]. Positive and negative body image operate on separate continuums, meaning interventions aimed at reducing body image distress do not necessarily promote positive body image, but instead may lead to neutral body image, limiting the ability to flourish [34]. The few studies that have investigated the impact of interventions on positive outcomes in those living with cancer were designed to promote self‐compassionate attitudes and implemented with breast cancer survivors only [35, 36].

Furthermore, interventions are often implemented after individuals have undergone treatment (instead of being offered *during* treatment as well) [10, 37, 38], and therefore after potential negative changes to body image have occurred. This gap in the standard of care during the treatment itself is consistent with previous research [39] and was reiterated to us during consultation with Wellspring members and administrators during the adaptation of this intervention, where many stated this was the first body image support they had received during their cancer journey. Given the benefits associated with positive body image and its likely protective nature for mental and physical health [34], there is a need for interventions that (1) specifically address how to facilitate positive body image and (2) are applicable to a diverse group of individuals living with diverse types of cancer.

Body Image Awareness Seminars (BIAS) is a unique positive body image program, created, using action research, with and for a heterogenous sample of older adults and individuals with cardiovascular disease, multiple sclerosis, and spinal cord injury [40]. Each of the program's six seminars has a unique goal centered around improvement of positive body image characteristics including but not limited to defining positive body image, social influences (e.g., media), coping strategies (e.g., media literacy), and mindfulness. The program includes education, guided activities, and facilitated discussion. Quantitative and qualitative findings showed body appreciation, satisfaction with body function, and intuitive eating all improved after the 6‐week program and were sustained at follow‐up. Researchers recommended examining the transferability of BIAS in new populations and settings [40].

Therefore, the purpose of the current study was to examine the impact of an adapted BIAS program on body image distress, body appreciation, functionality appreciation, body apperception, appearance evaluation, and self‐compassion in individuals living (in treatment, recovery, or any stage of diagnosis) with cancer. It was hypothesized that participants would experience significant improvements in both positive and negative body image variables, which would be sustained at follow‐up, consistent with the first iteration.

## 2 Methods

### 2.1 Study Context

This study took place with Wellspring members from across Canada. Wellspring is a cancer support center that provides free social, emotional, psychological, informational, and spiritual support care programs to individuals living with cancer (at any point in their journey including at diagnosis, during treatment, or post‐treatment), their families, and supporters. It offers a variety of supportive care programs (e.g., managing difficult symptoms of cancer or its treatment [e.g., nausea, hair loss, weight fluctuations, depression, anxiety, social isolation]), helping with cancer recovery and restoring health and wellness, transitioning back into the community, and managing health on a long‐term basis. Although medical referrals are not mandatory, many individuals discover Wellspring services through their healthcare providers and regional cancer centers. Additionally, Wellspring actively engages with the community to raise awareness about their mission through social media campaigns and a variety of outreach initiatives and promotional events, ensuring that more people become informed about the free cancer supportive care offered. With no existing program at Wellspring related to body image, it presented an ideal opportunity to meet the needs of the members and test the applicability of the BIAS program.

At the time of project implementation (February/March 2021), the COVID‐19 pandemic and frequent provincial public health restrictions meant in‐person programming was not taking place at Wellspring (given the health risks for those living with cancer). Although this meant the program had to be delivered online, it created the opportunity to deliver it across Canada.

### 2.2 Study Design

The project used a single‐group pretest–posttest design with a 6‐week follow‐up. This was only the second iteration of BIAS and the first time implementing the program in a new population and exclusively online. Given the exploratory nature of the study, it was important to determine the utility of BIAS in this group and format before the use of a randomized controlled trial model.

This study took place across two phases: adaptation of the program for those living with cancer and online delivery (through consultation with Wellspring members and administration; phase 1; reported elsewhere in MaGee & Gammage, in progress) and delivery of the BIAS program and evaluation of changes to body image (phase 2). Briefly, phase 1 used action research to adapt BIAS and improve the relevance and appropriateness of the program for this group, while adhering to the tenets of positive body image. This adapted program was delivered in the current study.

### 2.3 Participants

Clearance was obtained from the university ethics board (Brock REB file #20–201; January 2021) and Wellspring's Centre of Innovation (February 2021); participants were then recruited by Wellspring Niagara's Centre Manager who sent out information on the study to the organization's listserv and posted on their social media pages, inviting individuals to contact the researchers if interested. Using purposive sampling, 38 individuals signed up for the program. Participants took part in one of three 6‐week sessions based on their availability (May 12–June 16, 2021, May 14–June 18, 2021, and July 14–August 25, 2021). One participant withdrew to begin treatment again. Some participants were attending other Wellspring programs at the same time as BIAS took place. This may represent a potential confounder; however, there are no existing programs at Wellspring related to body image, reducing this possible influence.

Attendance to the program sessions (outside of data collection) was 100% and 76% for each of the May cohorts and 65% for the July cohort; average attendance was 80% across all cohorts. All attendance rates were higher than typical Wellspring programs (which report a 41%–43% dropout rate in multi‐week programs). A total of 26 participants provided complete data pre‐ and post‐intervention, with 11 providing complete data across all three time points (pre, post, 6 weeks post). Incomplete sets of data may have been in part due to anonymous IDs being entered incorrectly by participants (meaning they could not be matched for analysis), and the completely virtual experience (meaning requests for data collection were sent via email blasts with reliance on participants to receive and complete measures in a timely manner without in‐person oversight).

### 2.4 Materials

#### 2.4.1 Demographics

Participants self‐reported age, gender, ethnicity, occupation, stage of cancer journey, years living with cancer, type of cancer, and involvement in Wellspring (years and type of program involvement).

#### 2.4.2 Body Image Distress

The 10‐item Body Image Scale [17] was used to examine body image distress/disturbance specific to a cancer population. The measure was developed in a broad range of cancer patients; evidence of reliability, validity, and scale structure was provided [17]. Internal consistency reliability for the present study was satisfactory for baseline (*α* = 0.92), post (*α* = 0.92), and follow‐up (*α* = 0.84).

#### 2.4.3 Body Appreciation

The 10‐item Body Appreciation Scale‐2 [41] assesses acceptance and/or favorable opinions towards the body. This scale has demonstrated unidimensional structure, internal consistency, test–retest reliability, and construct validity in Western men and women from university and community samples [41] with satisfactory internal consistency reliability with breast cancer survivors [42, 43]. Internal consistency reliability for the present study was satisfactory for baseline (*α* = 0.95), post (*α* = 0.95), and follow‐up (*α* = 0.96).

#### 2.4.4 Functionality Appreciation

The 7‐item Functionality Appreciation Scale [44] measures the degree to which an individual appreciates, respects, and honors their body for what it is capable of doing. It has demonstrated high internal consistency reliability in samples of women with breast cancer [45]. Internal consistency reliability for the present study was satisfactory for baseline (*α* = 0.90), post (*α* = 0.88), and follow‐up (*α* = 0.94).

#### 2.4.5 Body Apperception

The 8‐item Measure of Body Apperception [19] measures the importance of perceptions of physical appearance and a sense of body integrity for self‐worth. This measure has shown adequate internal consistency and temporal stability in those living with early‐stage breast cancer, women pre‐surgery for breast cancer, and individuals living with head and neck cancer [46, 47]. Internal consistency reliability for the present study was generally satisfactory for the appearance investment subscale (baseline *α* = 0.76; post *α* = 0.65; follow‐up *α* = 0.71) and the body integrity subscale (baseline *α* = 0.65; post *α* = 0.60; follow‐up *α* = 0.75).

#### 2.4.6 Appearance Evaluation

The 7‐item Appearance Evaluation subscale from the Multidimensional Body‐Self Relations Questionnaire [48] was used to assess appearance satisfaction. The subscale has demonstrated acceptable reliability and validity in diverse samples, including older adults and those living with chronic disease [40, 49]. Internal consistency reliability for the present study was satisfactory for baseline (*α* = 0.85), post (*α* = 0.88), and follow‐up (*α* = 0.92).

#### 2.4.7 Self‐Compassion

The 12‐item Self‐Compassion Scale‐Short Form [50] assesses the ability to be with feelings of suffering in a warm and caring way. The short form has demonstrated good test–retest reliability and validity in women with breast cancer [51] and good internal consistency and test–retest reliability in clinical samples (those with psychiatric illness [52]). Internal consistency reliability for the present study was satisfactory for baseline (*α* = 0.85), post (*α* = 0.90), and follow‐up (*α* = 0.87).

### 2.5 Procedures

Twelve to 14 members were enrolled in each of the sessions based on recommendations by Bailey et al. (2019) and Wellspring administrators. This number allowed for participation by all members. The first author facilitated all seminars, while a research assistant took notes and monitored the “chat” function on Zoom. A Wellspring administrator attended every session to troubleshoot technological issues or in case a participant required support.

After group introductions during the first seminar (but before any seminar content), participants provided informed consent and completed the pre‐program questionnaire package on Qualtrics. The same process was completed in seminar 6 after all program content was delivered. Six weeks after the conclusion of the program, participants were emailed the same questionnaire package.

#### 2.5.1 Statistical Methods

##### 2.5.1.1 Data Preparation

All data were analyzed using SPSS 29.0. Data were screened for missing and inaccurate values by examining frequencies. Less than 5% of the data was missing, and values were replaced with a series mean. One outlier was identified (*z*‐score = −3.45, *p* < 0.001, two‐tailed test) and was winsorized. All data met statistical assumptions except for sphericity (investment in appearance subscale).

Means and standard deviations were calculated for all dependent variables, and bivariate correlations were calculated between demographic information and each of the dependent variables to identify potential covariates (none identified).

##### 2.5.1.2 Hypothesis Testing

Given the exploratory nature of the study, and consistent with Bailey et al. (2019), a series of paired sample *t*‐tests were conducted to examine changes in body image from baseline to post‐intervention. As an exploratory analysis, a series of one‐way repeated measures Analysis of Variance (ANOVA) tests were conducted to compare baseline, post‐intervention, and 6‐week follow‐up scores for the subsample of 11 participants with complete sets of data. To examine the nature of time effects, pairwise comparisons were completed (baseline versus post‐intervention, post‐intervention versus 6‐week follow‐up). For investment in appearance, the Greenhouse–Geisser correction is reported as the assumption of sphericity was violated (*p* < 0.05).

## 3 Results

With respect to the 37 participants (35 female, 2 male) who enrolled in the program, they ranged in age from 31 to 75 years (*M* = 56.13, SD = 9.63) and had been living with cancer anywhere from 3 months to 31 years. Of these 37 participants, 26 of them (25 female, 1 male) had data that could be matched pre‐ and post‐program (age *M* = 55.40, SD = 10.33). Table 1 presents a summary of sociodemographic characteristics of these 26 participants whose data were used in subsequent analyses.

**TABLE 1:** Summary of sociodemographic characteristics of 26 participants.

Characteristic	Frequency	%
Age		
< 50 (early onset)	5	19.2
50+	20	76.9
Cancer type		
Breast	17	63
Bone	1	3.7
Brain	1	3.7
Cervical	1	3.7
Myeloma	1	3.7
Neoplasm	1	3.7
Ovarian	1	3.7
Pancreatic	1	3.7
Bone sarcoma	1	3.7
Soft tissue sarcoma	1	3.7
Rectal	1	3.7
Treatment status		
In treatment (including pharmacological, chemotherapy, hormone, and radiation)	7	25.9
In remission	19	70.4

*Note:* Some participants were living with multiple types of cancer, meaning frequency counts will be greater than the sum of individual participants.

Results from paired sample *t*‐tests examining changes in body image pre‐to‐post program revealed significant differences for all variables: body image distress/disturbance (*t*_25_ = 5.01, *p* < 0.001), body appreciation (*t*_25_ = −4.01, *p* < 0.001), functionality appreciation (*t*_25_ = −2.82, *p* < 0.05), investment in appearance (*t*_25_ = 4.39, *p* < 0.001), body integrity (*t*_25_ = 4.70, *p* < 0.001), appearance evaluation (*t*_25_ = −4.70, *p* < 0.001), and self‐compassion (*t*_25_ = −5.75, *p* < 0.001). Examination of means (see Table 2) showed significant decreases in body image distress and disturbance, investment in appearance, and investment in body integrity, and significant increases in body appreciation, functionality appreciation, appearance evaluation, and self‐compassion.

**TABLE 2:** Means and standard deviations of measures pre‐and post‐program (*n* = 26).

Variable	Pre	Post
	*M* (SD)	*M* (SD)
Body Image Scale	28.04 (8.25)_a_	21.08 (7.27)_b_
Body Appreciation Scale‐2	3.13 (0.84)_a_	3.77 (0.69)_b_
Functionality Appreciation Scale	4.01 (0.77)_a_	4.42 (0.51)_b_
Measure of Body Apperception—Appearance Investment	10.20 (3.03)_a_	7.69 (2.36)_b_
Measure of Body Apperception—Body Integrity	10.06 (3.10)_a_	8.00 (2.45)_b_
Appearance Evaluation	2.67 (0.85)_a_	3.27 (0.82)_b_
Self‐Compassion Scale	2.75 (0.70)_a_	3.45 (0.77)_b_

*Note:* Different subscripts in each row indicate a significant difference (*p < 0.05*). Body Image Scale and Measure of Body Apperception range from 10 to 40, where lower scores indicate less negative body image. All remaining scales range from 1 to 5, where higher scores indicate a more positive body image.

Repeated measures ANOVAs for the exploratory analysis at follow‐up revealed a significant main effect for time for body image distress and disturbance *F* (2, 10) = 14.32, *p* < 0.001, η_p_^2^ = 0.589, body appreciation *F* (2, 10) = 16.10, *p* < 0.001, η_p_^2^ = 0.617, functionality appreciation *F* (2, 10) = 13.97, *p* < 0.001, η_p_^2^ = 0.583, investment in appearance *F* (1.29, 10) = 5.07, *p* < 0.05, η_p_^2^ = 0.337, investment in body integrity *F* (2, 10) = 8.97, *p* < 0.01, η_p_^2^ = 0.473, appearance evaluation *F* (2, 10) = 14.41, *p* < 0.001, η_p_^2^ = 0.590, and self‐compassion *F* (2, 10) = 5.99, *p* < 0.01, η_p_^2^ = 0.375, with all effects large. Pairwise comparisons revealed significant increases (see Table 3) from baseline to post‐intervention (*p*s < 0.05) for all measures (except investment in appearance), with changes sustained at follow‐up (*p*s > 0.05). For appearance investment, there was no difference at either time point.

**TABLE 3:** Means and standard deviations of measures pre, post, and 6 weeks following program (*n* = 11).

Variable	Pre	Post	Follow‐up
	*M* (SD)	*M* (SD)	*M* (SD)
Body Image Scale	26.91 (9.07)_a_	17.27 (4.86)_b_	16.64 (4.63)_b_
Body Appreciation Scale‐2	3.05 (0.82)_a_	4.10 (0.56)_b_	4.11 (0.63)_b_
Functionality Appreciation Scale	3.68 (0.74)_a_	4.64 (0.39)_b_	4.55 (0.53)_b_
Measure of Body Apperception—Appearance Investment	9.30 (3.06)	6.82 (2.27)	7.91 (2.17)
Measure of Body Apperception—Body Integrity	9.18 (3.46)_a_	7.18 (2.71)_b_	7.27 (2.41)_b_
Appearance Evaluation	2.70 (0.89)_a_	3.55 (0.77)_b_	3.44 (0.85)_b_
Self‐Compassion Scale	2.87 (0.56)_a_	3.59 (0.62)_b_	3.39 (0.70)_b_

*Note:* Different subscripts in each row indicate a significant difference (*p < 0.05*). Body Image Scale and Measure of Body Apperception range from 10 to 40, where lower scores indicate less negative body image, and individual items are summed. All remaining scales range from 1 to 5, where higher scores indicate more positive body image, and individual items are averaged.

## 4 Discussion

The purpose of this study was to examine the impact of the adapted BIAS program on the body image of individuals living with cancer. Consistent with hypotheses and previous evidence [40], participants reported significant improvements in positive body image (i.e., body appreciation, functionality appreciation) and negative body image (i.e., body image distress and disturbance, investment in body integrity, and appearance evaluation), which were sustained at follow‐up in a subsample of participants. Improvements were shown in both general and cancer‐specific measures of body image. These preliminary findings suggest that the adapted BIAS program can lead to improvements in both positive and negative body image in individuals living with diverse types of cancer.

The program's ability to both increase positive body image and decrease negative body image mirrored findings from Bailey et al. (2019), which was one of the first studies to demonstrate a program's ability to influence change on both body image continuums. Furthermore, consistent with Bailey et al. (2019), participants in our program also experienced improvement in self‐compassion. In our program, psychoeducation on the topic of self‐compassion and an expressive writing activity were specifically added during the adaptation phase of this project; (MaGee & Gammage, in progress), based on findings from Bailey et al. (2019) and consultation with members. Although distinct from positive body image, self‐compassion has been found to be positively related to body appreciation [53] and several interventions targeting self‐compassion in breast cancer survivors have led to improvements in body appreciation [42, 43]. These studies have tended to use self‐compassion‐based writing activities and have generally shown improvements such as less negative affect and body image‐related distress, and more self‐compassion and body appreciation following the interventions [54, 55]. Improvements in body image‐related distress occurred via improvements in self‐compassion [55]. Importantly, and of relevance to this study (delivered entirely by a researcher), benefits were achieved independently, without clinician support. Similarly, a recent review of nine compassion‐based interventions for cancer patients from randomized controlled trials found effectiveness in constructive compassion‐based interventions (multiple sessions with a theoretical basis) and brief compassion‐based interventions (3/4 interventions used writing guided by self‐compassionate prompts) with face‐to‐face and non‐face‐to‐face delivery formats being equally as effective in eliciting improvements in self‐compassion [56]. After synthesizing these findings [54, 55, 56], there appears to be consistency in the effectiveness of expressive writing activities for improving self‐compassion in a cancer population. The current study adds to this body of research by demonstrating improvements in a diverse sample (beyond just breast cancer or an otherwise homogeneous sample [56]) and beyond primarily writing interventions. The addition of a brief self‐compassion intervention was ideal for our program as it allowed the focus on positive body image to be maintained while targeting an additional variable known to facilitate improvements in body appreciation [42, 43]; this dyadic model may be beneficial to future facilitators of body image–focused interventions. Furthermore, greater self‐compassion is associated with a decrease in psychopathological symptoms, depression, and anxiety, and an increase in quality of life of cancer patients [57] which complements the goals of programs such as BIAS. Finally, the proven effectiveness of web‐based delivery (asynchronous) formats identified by Fan et al. (2023) is consistent with the results of the current study and emerging research [58]. For individuals still receiving medical treatment, the convenience of online approaches may be more feasible than traveling to and attending face‐to‐face sessions [58]. This delivery format also allowed us to reach participants nationally in the current study, broadening the impact of our program.

Before BIAS, there have been several interventions designed to improve body image in cancer survivors, which has generally been conceptualized as reducing negative body image. A review by Lewis‐Smith (2018) summarized findings from 26 body image interventions among women treated for breast cancer, measuring changes in body image distress, sadness due to hair loss, strength and health, social barriers, appearance and sexuality, and self‐concept [59]. Effective interventions (defined as significant improvements in body image variables at posttest and/or at follow‐up among the intervention group, relative to the control group) adopted either a psychoeducational (e.g., knowledge about the condition and coping skills) or psychotherapeutic (e.g., cognitive behavioral therapy) approach. Findings from our study mirror the effectiveness of multi‐session, group‐based interventions identified in this review, while contrasting findings regarding components and content of the effective interventions. For example, Lewis‐Smith (2018) found that effective psychoeducational interventions focused on identifying and managing stressors and symptoms, managing changes to the body and sexuality, information and support on the disease, surgery, and aftercare, and expected appearance changes after surgical procedures [59]. Given the specificity of these topics to individual circumstances and specific diagnoses, it limits their inclusivity. In fact, the authors of the review recommended that future research aim to shift the approach of interventions for this population from treatment‐focused to targeting broader modifiable influences on body image.

BIAS meets this call for action as it has now demonstrated in two diverse populations with diverse health conditions, its ability to improve body image [40]. The benefit of its applicability is that it can reach more individuals to evoke change in those living with the poor psychological outcomes associated with negative body image. Furthermore, aspects of body image that were improved in previous studies (e.g., dissatisfaction with appearance and scarring) were exclusive to reducing negative body image [38]. Researchers have argued that only focusing on alleviating negative body image and its pathology without considering how to promote positive body image has limited our understanding of the body image construct as a whole [34, 60, 61]. Tylka and Wood‐Barcalow (2015) highlight the potential harm of this approach, stating that “if body image therapies reduce symptoms of negative body image, but do not enhance aspects of positive body image, they may promote a neutral body image at best (e.g., ‘I don't hate my body anymore. I merely tolerate it.’; p. 118).”

Even most recently in a systematic review aimed at improving the understanding of the concept of body image in the context of psychosocial interventions for breast cancer patients [33], the authors noted that out of 22 interventions, body image was primarily considered in terms of body dissatisfaction and alleviating symptoms of negative body image without considering potential dimensions of positive body image. In fact, only three studies measured body appreciation as a notable outcome of interest [54, 55, 62]. The impact of BIAS on both negative and positive body image continuums improves the likelihood of flourishing in participants by moving beyond neutral body image into the fostering of protective qualities for mental and physical health [34]. Interestingly, there were no interventions identified by Lewis‐Smith (2018) with improvements at both post‐intervention *and* follow‐up [59]. The improvements found in the current study were sustained at follow‐up in a subsample of participants, consistent with Bailey et al. (2019). Our findings show the ability of BIAS to facilitate longer‐term improvements in the body image of individuals living with cancer and support its ability to inform healthcare provision and strategic directions for research.

Of the interventions aimed to improve positive body image (outside populations living with cancer), most have generally been created for, and tested with, adolescent or young adult women [63, 64, 65] and more diverse populations (e.g., men, people living with chronic disease or disability) are underrepresented [66]. The first iteration of BIAS addressed this underrepresentation in positive body image research (and body image intervention research more generally) by being one of the first programs to be created by, and implemented for, a diversity of populations including those with physical disability and/or chronic health conditions. Bailey et al. (2019) found that participants reported diversity in terms of gender and ability to be a strength of the program, as they gained varying perspectives on positive body image and challenged stereotypes they held. This also highlights the benefits of group‐based programs. Of the limited interventions effective in improving positive body image in adults living with a chronic disease, the most successful has been an individual, online writing‐based functionality intervention [66]. The current study shows the effectiveness of a multidimensional, group psychosocial approach to improve multiple components of both positive and negative body image in adults living with cancer. BIAS uses cognitive‐behavioral techniques and psychoeducation, approaches that have previously led to improvements in components of positive body image in non‐cancer populations [63, 65]. The group‐based approach was particularly advantageous, effective, and desirable in the current study given our population of interest. Wellspring operates under a peer‐support mandate providing programs that are designed to provide connection and belonging, meaning group support is a critical part of, and foundation to, their programs. As such, this study not only supports BIAS' ability to cultivate positive body image qualities in those living with cancer but also contributes to the literature on the effectiveness of cognitive behavioral therapy and psychoeducation using approaches that highlight body functionality as promising techniques for improving body image [67, 68, 69].

This iteration of BIAS was able to provide significant contributions to body image intervention research in multiple ways. The improvement in body and functionality appreciation found in the current study provides additional support for the universality of many positive body image characteristics (e.g., acceptance and gratitude for what the body can do) previously reported in adolescents and young adults, people living with spinal cord injury, women with rheumatoid arthritis, and women with multiple sclerosis [70, 71, 72]. However, this study also supports the need to acknowledge nuances in the different body image experiences across social identities [73, 74]. This delicate balance between the consistency of basic body image concepts with nuances specific to one's social identity [74] was the main motivation for the adaptation phase of this study. During this phase, certain aspects of the BIAS program were revised to improve the relevance and appropriateness of the program to those living with cancer (e.g., removal of example making light of hair loss), while the overall tenets of BIAS and positive body image were maintained. Our action research approach was critical to the findings in phase 1. Action research principles were applied in both iterations of BIAS as a means of bridging the gap between theory and real‐life experiences [75]. Action research is an ideal method of inquiry from the standpoint of complex and nuanced body image experiences [76]. Program adaptations occurred alongside a key group of end users (i.e., those living with cancer) who provided “insider” information (e.g., body image outcomes during cancer treatment and recovery) that improved the relevance of our program and contributed to the desired change in participants [75].

The current study successfully addressed several recommendations from previous work in the field, aiding in the advancement of research on effective positive body image interventions. Guest et al. (2019) urged researchers to deliver interventions that incorporate approaches from existing interventions to target and promote multiple components of positive body image as a means of capitalizing on its protective mechanisms for mental and physical health and link to adaptive outcomes [34]. The foundation of BIAS is positive body image research and theory, where each unique tenet was incorporated into education, activities, and/or discussion. Each session has a unique theme designed to promote respect, acceptance, and appreciation for the body, including definitions and research about positive body image, education on the impact of social influences (including media literacy) on body image, body image coping strategies, individual differences in body image including learning to conceptualize beauty more broadly, and respecting the body including mindfulness. As such, the current study found improvements in the body image of participants but also met the need for an intervention specifically designed around positive body image tenets. BIAS also utilizes behavioral techniques and psychoeducation approaches that have previously been successful [66]. Recommendations from Brunet et al. (2024) to improve interventions in women undergoing or following treatment for breast cancer were also achieved by explicitly and exclusively addressing body image in intervention work [77], addressing broader sociocultural and psychological influences on body image, rather than taking a narrow disease‐focused approach, and using follow‐up evaluations and tracking attrition reasons and rates.

It is important to acknowledge the implications of this iteration's online delivery. The current study reported improvements consistent with the original, in‐person program [40]. The ability to offer the program online, without compromising its impact, means BIAS can be delivered in various formats to improve accessibility and potential adherence. This is particularly relevant for those who experience fluctuations in their health status, challenges related to accessibility, or who are otherwise unable to commit to in‐person sessions. The sustained impact of changes 6 weeks post‐program also improved the long‐term sustainability of positive body image outcomes shown in previous online body image programs (e.g., 1‐month post‐program) [66, 78].

### 4.1 Study Limitations and Future Research

There are some limitations of the current study. Although there was diversity in participants in terms of age, type, and stage of cancer, there was a lack of diversity in race and ethnicity (majority of participants were White), and only two were men. Future facilitators, using their unique insider knowledge of their end‐users, should consider how to increase the number of men who participate in the program (e.g., hand out program materials to male‐only groups to advertise, spark interest, and reduce barriers/stigma); this approach could also be successful in diversifying participation in terms of other characteristics (e.g., race/ethnicity). Despite the superior enrollment and adherence to our program compared to typical Wellspring programs, adherence to data collection was not robust, limiting the power of our statistical analyses. Finally, the lack of a control group (given the exploratory nature of this study) limits the conclusions that can be drawn.

Given the success in delivering BIAS in an online format for those living with cancer, a randomized controlled trial (with adequate sample size) should be conducted to identify causal relationships. Our small sample size limited our ability to examine the effectiveness of the program across a range of factors such as age, diagnosis stage, and cancer type, and future research should examine whether this program is more beneficial for some groups than others. Further, future studies should examine how these factors may have implications for the delivery of the program to those living with cancer, to continue enhancing its success (e.g., supporting Information specific to treatment type). Future studies with other populations will need to continue the consultation process with end users (e.g., schools, rehabilitation centers, community centers) to determine any necessary adaptations in examples, activities, or discussion; this preliminary step was essential to the success of BIAS in this study by ensuring relevance and sensitivity to the specific population being investigated while maintaining adherence to the broad principles of the program.

### 4.2 Clinical Implications

Our participants reported a notable lack of conversation around body image in their standard of care while living with cancer. Future collaborations with healthcare providers could allow for BIAS to be integrated into support services for this population. Furthermore, studies have shown that psychological distress among survivors of various cancer types and in various stages of cancer, including long‐term survivorship, is higher compared with matched controls [79]. This provides support for the fact that the psychological adjustment (including body image–related concerns) of the cancer experience extends far beyond treatment or periods of stability. There is evidence to substantiate the need for the standard implementation of an intervention such as BIAS during or post‐treatment for those living with cancer. Given that the BIAS manual was designed so that non‐body image experts can facilitate the program, there are implications for BIAS to be implemented in the real world, with minimal barriers (facilitators could include psychologists, counselors), to continue to affect positive change.

## 5 Conclusion

The BIAS program was associated with increases in positive body image and reductions in negative body image outcomes in both men and women of varying ages living with different types of cancer. This study addressed the need for an intervention that facilitates positive body image outcomes in a diverse group of individuals living with cancer across Canada. Future large‐scale randomized controlled trials should be undertaken to test its effectiveness and improve an important psychological dimension of the cancer experience.

## Author Contributions

**Carly A. MaGee:** conceptualization, data curation, formal analysis, investigation, methodology, project administration, resources, validation, visualization, writing – original draft. **Kimberley L. Gammage:** conceptualization, methodology, resources, supervision, validation, writing – review and editing.

## Conflicts of Interest

The authors declare no conflicts of interest.

## Data Availability

The data that support the findings of this study are available from the corresponding author upon reasonable request.

## References

[1] E. Byrne , J. Gaffey , L. Hayden , A. Daly , P. Gallagher , and S. Dunne , “Body Image and Cancer‐Related Lymphoedema: A Systematic Review,” Psycho‐Oncology 32, no. 10 (2023): 1528–1538. 37681525 10.1002/pon.6215

[2] C. Davis , P. Tami , D. Ramsay , et al., “Body Image in Older Breast Cancer Survivors: A Systematic Review,” Psycho‐Oncology 29, no. 5 (2020): 823–832. 32048373 10.1002/pon.5359

[3] V. D. Phung and S. Y. Fang , “Body Image Issues in Patients With Colorectal Cancer a Scoping Review,” Cancer Nursing 46, no. 3 (2023): 233. 35349543 10.1097/NCC.0000000000001085

[4] M. Fardouly , V. Kakar , and P. C. Diedrichs , “Routledge Handbook of Gender and Communication,” in Body Image and Global Media (Routledge, 2021).

[5] H. Lewis‐Smith , P. C. Diedrichs , and E. Halliwell , “Cognitive‐Behavioral Roots of Body Image Therapy and Prevention,” Body Image 31 (2019): 309–320. 31519523 10.1016/j.bodyim.2019.08.009

[6] M. Massey‐Stokes , M. Golman , A. Quezada Ochoa , A. Stokes , and J. J. Robert‐McComb , “Body Image Throughout the Lifespan,” in Body Image Throughout the Lifespan, 3rd ed. (Springer International Publishing, 2023), 30.

[7] M. A. Ellis , K. R. Sterba , E. A. Brennan , et al., “A Systematic Review of Patient‐Reported Outcome Measures Assessing Body Image Disturbance in Patients With Head and Neck Cancer,” Journal of Otolaryngology ‐ Head & Neck Surgery 160, no. 6 (2019): 941–954. 10.1177/0194599819829018 PMC6546516 30744514

[8] P. A. Parker , A. Youssef , S. Walker , et al., “Short‐Term and Long‐Term Psychosocial Adjustment and Quality of Life in Women Undergoing Different Surgical Procedures for Breast Cancer,” Annals of Surgical Oncology 14 (2007): 3078–3089. 17574501 10.1245/s10434-007-9413-9

[9] M. F. Vani , K. M. Lucibello , L. Trinh , D. Santa Mina , and C. M. Sabiston , “Body Image Among Adolescents and Young Adults Diagnosed With Cancer: A Scoping Review,” Psycho‐Oncology 30, no. 8 (2021): 1278–1293. 33882162 10.1002/pon.5698

[10] C. L. Paterson , C. A. Lengacher , K. A. Donovan , K. E. Kip , and C. S. Tofthagen , “Body Image in Younger Breast Cancer Survivors: A Systematic Review,” Cancer Nursing 39, no. 1 (2016): E39–E58. 10.1097/NCC.0000000000000251 PMC4607543 25881807

[11] M. Henry , J. G. Albert , S. Frenkiel , et al., “Body Image Concerns in Patients With Head and Neck Cancer: A Longitudinal Study,” Frontiers in Psychology 13, no. 3 (2022): 816587. 35401366 10.3389/fpsyg.2022.816587 PMC8988682

[12] A. Begovic‐Juhant , A. Chmielewski , S. Iwuagwu , and L. A. Chapman , “Impact of Body Image on Depression and Quality of Life Among Women With Breast Cancer,” Journal of Psychosocial Oncology 30, no. 4 (2012): 446–460. 22747107 10.1080/07347332.2012.684856

[13] T. S. Guedes , N. P. de Oliveira , A. M. Holanda , et al., “Body Image of Women Submitted to Breast Cancer Treatment,” Asian Pacific Journal of Cancer Prevention 19, no. 6 (2018): 1487. 29936719 10.22034/APJCP.2018.19.6.1487 PMC6103585

[14] C. D. Runowicz , C. R. Leach , N. L. Henry , et al., “American Cancer Society/American Society of Clinical Oncology Breast Cancer Survivorship Care Guideline,” Journal of Clinical Oncology 34, no. 6 (2016): 611–635. 26644543 10.1200/JCO.2015.64.3809

[15] C. A. Falk Dahl , K. V. Reinertsen , I. L. Nesvold , S. D. Fosså , and A. A. Dahl , “A Study of Body Image in Long‐Term Breast Cancer Survivors,” Cancer 116, no. 15 (2010): 3549–3557. 20564138 10.1002/cncr.25251

[16] P. Hopwood , “The Assessment of Body Image in Cancer Patients,” European Journal of Cancer 29, no. 2 (1993): 276–281. 10.1016/0959-8049(93)90193-j 8422297

[17] P. Hopwood , I. Fletcher , A. Lee , and S. al Ghazal , “A Body Image Scale for Use With Cancer Patients,” European Journal of Cancer 37, no. 2 (2001): 189–197. 11166145 10.1016/s0959-8049(00)00353-1

[18] N. N. Baxter , P. J. Goodwin , R. S. Mcleod , R. Dion , G. Devins , and C. Bombardier , “Reliability and Validity of the Body Image After Breast Cancer Questionnaire,” Breast Journal 12, no. 3 (2006): 221–232. 16684320 10.1111/j.1075-122X.2006.00246.x

[19] C. S. Carver , C. Pozo‐Kaderman , A. A. Price , et al., “Concern About Aspects of Body Image and Adjustment to Early Stage Breast Cancer,” Psychosomatic Medicine 60, no. 2 (1998): 168–174. 9560865 10.1097/00006842-199803000-00010

[20] T. L. Fazzino , R. C. Hunter , N. Sporn , D. N. Christifano , and C. A. Befort , “Weight Fluctuation During Adulthood and Weight Gain Since Breast Cancer Diagnosis Predict Multiple Dimensions of Body Image Among Rural Breast Cancer Survivors,” Psycho‐Oncology 26, no. 3 (2017): 392–399. 26564108 10.1002/pon.4035 PMC5568609

[21] M. C. Fingeret , I. Teo , and D. E. Epner , “Managing Body Image Difficulties of Adult Cancer Patients: Lessons From Available Research,” Cancer 120, no. 5 (2014): 633–641. 10.1002/cncr.28469 PMC4052456 24895287

[22] J. M. Harrington , E. G. Jones , and T. Badger , “Body Image Perceptions in Men With Prostate Cancer,” In Oncology Nursing Forum 36, no. 2 (2009): 167–172. 10.1188/09.ONF.167-172 19273405

[23] K. Zhou , X. He , L. Huo , et al., “Development of the Body Image Self‐Rating Questionnaire for Breast Cancer (BISQ‐BC) for Chinese Mainland Patients,” BMC Cancer 18 (2018): 1–8. 29301503 10.1186/s12885-017-3865-5 PMC5753569

[24] V. Sebri and G. Pravettoni , “Tailored Psychological Interventions to Manage Body Image: An Opinion Study on Breast Cancer Survivors,” International Journal of Environmental Research and Public Health 20, no. 4 (2023): 2991. 36833684 10.3390/ijerph20042991 PMC9957299

[25] W. W. Lam , M. Ye , and R. Fielding , “Trajectories of Quality of Life Among Chinese Patients Diagnosed With Nasopharynegeal Cancer,” PLoS One 7, no. 9 (2012): e44022. 23028484 10.1371/journal.pone.0044022 PMC3445583

[26] F. Cousson‐Gelie , M. Bruchon‐Schweitzer , J. M. Dilhuydy , and M. A. Jutand , “Do Anxiety, Body Image, Social Support and Coping Strategies Predict Survival in Breast Cancer?,” A Ten‐Year Follow‐Up Study. Psychosomatics 48, no. 3 (2007): 211–216. 17478589 10.1176/appi.psy.48.3.211

[27] S. M. Rosenberg , R. M. Tamimi , S. Gelber , et al., “Body Image in Recently Diagnosed Young Women With Early Breast Cancer,” Psycho‐Oncology 22, no. 8 (2013): 1849–1855. 23132765 10.1002/pon.3221 PMC3594059

[28] P. Kenny , M. T. King , A. Shiell , et al., “Early Stage Breast Cancer: Costs and Quality of Life One Year After Treatment by Mastectomy or Conservative Surgery and Radiation Therapy,” Breast 9, no. 1 (2000): 37–44. 14731583 10.1054/brst.1999.0111

[29] K. D. Miller , R. L. Siegel , C. C. Lin , et al., “Cancer Treatment and Survivorship Statistics, 2016,” CA: a Cancer Journal for Clinicians 66, no. 4 (2016): 271–289. 27253694 10.3322/caac.21349

[30] E. Gilbert , J. M. Ussher , and J. Perz , “Renegotiating Sexuality and Intimacy in the Context of Cancer: The Experiences of Carers,” Archives of Sexual Behavior 39 (2010): 998–1009. 19067153 10.1007/s10508-008-9416-z

[31] E. M. Graboyes , S. Maurer , Y. Park , et al., “Evaluation of a Novel Telemedicine‐Based Intervention to Manage Body Image Disturbance in Head and Neck Cancer Survivors,” Psycho‐Oncology 29, no. 12 (2020): 1988–1994. 32350999 10.1002/pon.5399 PMC7606304

[32] L. Trachtenberg , J. Wong , H. Rennie , et al., “Feasibility and Acceptability of i‐Restoring Body Image After Cancer (i‐ReBIC): A Pilot Trial for Female Cancer Survivors,” Psycho‐Oncology 29, no. 4 (2020): 639–646. 31724261 10.1002/pon.5288

[33] L. Anne Thamar Louis , J. Fortin , C. A. Roy , A. Brunet , and A. Aimé , “Body Image Interventions Within Breast Cancer Care: A Systematic Review and Concept Analysis,” Journal of Psychosocial Oncology 42, no. 3 (2024): 427–447. 37609854 10.1080/07347332.2023.2249879

[34] T. L. Tylka and N. L. Wood‐Barcalow , “What Is and What Is Not Positive Body Image? Conceptual Foundations and Construct Definition,” Body Image 14 (2015): 118–129. 25921657 10.1016/j.bodyim.2015.04.001

[35] Y. Chen , R. Liu , J. Xiao , et al., “Effects of Online Mindful Self‐Compassion Intervention on Negative Body Image in Breast Cancer Patients: A Randomized Controlled Trail,” European Journal of Oncology Nursing 72 (2024): 102664. 39059197 10.1016/j.ejon.2024.102664

[36] H. Gopan , E. Rajkumar , A. Gopi , and J. Romate , “Mindfulness‐Based Interventions for Body Image Dissatisfaction Among Clinical Population: A Systematic Review and Meta‐Analysis,” British Journal of Health Psychology 29, no. 2 (2024): 488–509. 38097499 10.1111/bjhp.12710

[37] W. Zhao , Y. Y. Chong , and W. T. Chien , “Effectiveness of Cognitive‐Based Interventions for Improving Body Image of Patients Having Breast Cancer: A Systematic Review and Meta‐Analysis,” Asia‐Pacific Journal of Oncology Nursing 10, no. 4 (2023): 100213. 37089782 10.1016/j.apjon.2023.100213 PMC10120298

[38] H. Lewis‐Smith , P. C. Diedrichs , N. Rumsey , and D. Harcourt , “Efficacy of Psychosocial and Physical Activity‐Based Interventions to Improve Body Image Among Women Treated for Breast Cancer: A Systematic Review,” Psycho‐Oncology 27, no. 12 (2018): 2687–2699. 30161285 10.1002/pon.4870

[39] M. J. Esplen , E. Warner , V. Boquiren , J. Wong , and B. Toner , “Restoring Body Image After Cancer (ReBIC): A Group Therapy Intervention,” Psychooncology 4, no. 29 (2020): 671–680. 10.1002/pon.5304 31984589

[40] K. A. Bailey , K. L. Gammage , and C. van Ingen , “Designing and Implementing a Positive Body Image Program: Unchartered Territory With a Diverse Team of Participants,” Action Research 17, no. 2 (2019): 146–161.

[41] T. L. Tylka and N. L. Wood‐Barcalow , “The Body Appreciation Scale‐2: Item Refinement and Psychometric Evaluation,” Body Image 12 (2015): 53–67. 25462882 10.1016/j.bodyim.2014.09.006

[42] M. Ferrari , C. Hunt , A. Harrysunker , M. J. Abbott , A. P. Beath , and D. A. Einstein , “Self‐Compassion Interventions and Psychosocial Outcomes: A Meta‐Analysis of RCTs,” Mindfulness 10 (2019): 1455–1473.

[43] V. Sebri , I. Durosini , S. Triberti , and G. Pravettoni , “The Efficacy of Psychological Intervention on Body Image in Breast Cancer Patients and Survivors: A Systematic‐Review and Meta‐Analysis,” Frontiers in Psychology 12 (2021): 611954. 33732184 10.3389/fpsyg.2021.611954 PMC7957010

[44] J. M. Alleva , T. L. Tylka , and A. M. Van Diest , “The Functionality Appreciation Scale (FAS): Development and Psychometric Evaluation in US Community Women and Men,” Body Image 23 (2017): 28–44. 28822275 10.1016/j.bodyim.2017.07.008

[45] K. Ettridge , K. Scharling‐Gamba , C. Miller , D. Roder , and I. Prichard , “Body Image and Quality of Life in Women With Breast Cancer: Appreciating the Body and Its Functionality,” Body Image 40 (2022): 92–102. 34902783 10.1016/j.bodyim.2021.11.001

[46] P. Jean‐Pierre , C. Fundakowski , E. Perez , et al., “Latent Structure and Reliability Analysis of the Measure of Body Apperception: Cross‐Validation for Head and Neck Cancer Patients,” Supportive Care in Cancer 21 (2013): 591–598. 22886430 10.1007/s00520-012-1561-1 PMC4086048

[47] V. M. Petronis , C. S. Carver , M. H. Antoni , and S. Weiss , “Investment in Body Image and Psychosocial Well‐Being Among Women Treated for Early Stage Breast Cancer: Partial Replication and Extension,” Psychology & Health 18, no. 1 (2003): 1–3.

[48] T. F. Cash , “The Multidimensional Body Self‐Relations Questionnaire Users' Manual,” Research Gate [Internet] (2000): 1–12.

[49] J. Brunet and J. Price , “A Scoping Review of Measures Used to Assess Body Image in Women With Breast Cancer,” Psycho‐Oncology 30, no. 5 (2021): 669–680. 33480160 10.1002/pon.5619

[50] F. Raes , E. Pommier , K. D. Neff , and D. van Gucht , “Construction and Factorial Validation of a Short Form of the Self‐Compassion Scale,” Clinical Psychology & Psychotherapy 18, no. 3 (2011): 250–255. 21584907 10.1002/cpp.702

[51] N. Todorov , K. A. Sherman , C. J. Kilby , and Breast Cancer Network Australia , “Self‐Compassion and Hope in the Context of Body Image Disturbance and Distress in Breast Cancer Survivors,” Psycho‐Oncology 28, no. 10 (2019): 2025–2032. 31369188 10.1002/pon.5187

[52] S. Alfonsson , E. Winai , E. Collin , M. Isaksson , and M. Wolf‐Arehult , “The Self‐Compassion Scale–Short Form: Psychometric Evaluation in One Non‐Clinical and Two Clinical Swedish Samples,” Clinical Psychology & Psychotherapy 30, no. 3 (2023): 631–642. 36648383 10.1002/cpp.2830

[53] K. J. August , D. Malik , C. H. Markey , K. Woods , and G. C. Gerwitz , “Additive and Interactive Associations Among Body Appreciation, Self‐Compassion, and Gender in Understanding College Students' Health Behaviors,” Body Image 47 (2023): 101634. 37774424 10.1016/j.bodyim.2023.101634

[54] A. Przezdziecki and K. A. Sherman , “Modifying Affective and Cognitive Responses Regarding Body Image Difficulties in Breast Cancer Survivors Using a Self‐Compassion‐Based Writing Intervention,” Mindfulness 7 (2016): 1142–1155.

[55] K. A. Sherman , A. Przezdziecki , J. Alcorso , et al., “Reducing Body Image–Related Distress in Women With Breast Cancer Using a Structured Online Writing Exercise: Results From the My Changed Body Randomized Controlled Trial,” Journal of Clinical Oncology 36, no. 19 (2018): 1930–1940. 29688834 10.1200/JCO.2017.76.3318

[56] Y. C. Fan , F. H. Hsiao , and C. C. Hsieh , “The Effectiveness of Compassion‐Based Interventions Among Cancer Patients: A Systematic Review and Meta‐Analysis,” Palliative & Supportive Care 21, no. 3 (2023): 534–546. 36397274 10.1017/S1478951522001316

[57] J. Pinto‐Gouveia , C. Duarte , M. Matos , and S. Fráguas , “The Protective Role of Self‐Compassion in Relation to Psychopathology Symptoms and Quality of Life in Chronic and in Cancer Patients,” Clinical Psychology & Psychotherapy 21, no. 4 (2014): 311–323. 23526623 10.1002/cpp.1838

[58] J. Austin , M. J. Schroevers , J. Van Dijk , et al., “Compas‐Y: A Mixed Methods Pilot Evaluation of a Mobile Self‐Compassion Training for People With Newly Diagnosed Cancer,” Digital Health 9 (2023): 20552076231205272. 37868157 10.1177/20552076231205272 PMC10588427

[59] H. Lewis‐Smith , P. C. Diedrichs , N. Rumsey , et al., “Efficacy of Psychosocial and Physical Activity‐Based Interventions to Improve Body Image Among Women Treated for Breast Cancer: A Systematic Review,” Psycho‐Oncology 27, no. 12 (2018): 2687–2699. 30161285 10.1002/pon.4870

[60] K. A. Bailey , K. L. Gammage , C. van Ingen , and D. S. Ditor , “‘It's all About Acceptance’: A Qualitative Study Exploring a Model of Positive Body Image for People With Spinal Cord Injury,” Body Image 15 (2015): 24–34. 26002149 10.1016/j.bodyim.2015.04.010

[61] L. Smolak and T. F. Cash , Future Challenges for Body Image Science, Practice, and Prevention (Guilford Press, 2011).

[62] A. Mifsud , M. J. Pehlivan , P. Fam , et al., “Feasibility and Pilot Study of a Brief Self‐Compassion Intervention Addressing Body Image Distress in Breast Cancer Survivors,” Health Psychology and Behavioral Medicine 9, no. 1 (2021): 498–526. 34104572 10.1080/21642850.2021.1929236 PMC8158280

[63] A. C. Amaral , E. Stice , and M. E. Ferreira , “A Controlled Trial of a Dissonance‐Based Eating Disorders Prevention Program With Brazilian Girls,” Psicologia: Reflexão e Crítica 32, no. 13 (2019): 1–10. 10.1186/s41155-019-0126-3 PMC6967323 32026167

[64] E. Guest , H. Jarman , N. Sharratt , et al., “‘Everybody's Different: The Appearance Game’. A Randomised Controlled Trial Evaluating an Appearance‐Related Board Game Intervention With Children Aged 9–11 Years,” Body Image 36 (2021): 34–44. 33160256 10.1016/j.bodyim.2020.09.010

[65] C. Sundgot‐Borgen , O. Friborg , E. Kolle , et al., “The Healthy Body Image (HBI) Intervention: Effects of a School‐Based Cluster‐Randomized Controlled Trial With 12‐Months Follow‐Up,” Body Image 29 (2019): 122–131. 30928681 10.1016/j.bodyim.2019.03.007

[66] E. Guest , B. Costa , H. Williamson , J. Meyrick , E. Halliwell , and D. Harcourt , “The Effectiveness of Interventions Aiming to Promote Positive Body Image in Adults: A Systematic Review,” Body Image 30 (2019): 10–25. 31077956 10.1016/j.bodyim.2019.04.002

[67] J. Brooks , D. C. Walker , and K. Murray , “What Can My Body Do for Me? Guided Body‐Functionality Mirror Gazing Task Improved College Women's Body Appreciation and Body Functionality Orientation,” Journal of American College Health 3 (2023): 1–196. 10.1080/07448481.2023.2209195 37207308

[68] M. E. Rekkers , L. Aardenburg , M. Scheffers , A. A. van Elburg , and J. T. van Busschbach , “Shifting the Focus: A Pilot Study on the Effects of Positive Body Exposure on Body Satisfaction, Body Attitude, Eating Pathology and Depressive Symptoms in Female Patients With Eating Disorders,” International Journal of Environmental Research and Public Health 19, no. 18 (2022): 11794. 36142068 10.3390/ijerph191811794 PMC9517204

[69] D. C. Walker and K. Murray , “A Pilot Clinical Case Series of Functionality‐Focused Mirror Exposure in Women With Clinically Elevated Body Dissatisfaction,” Cognitive and Behavioral Practice 31, no. 1 (2024): 90–108.

[70] J. M. Alleva and T. L. Tylka , “Body Functionality: A Review of the Literature,” Body Image 36 (2021): 149–171. 33321273 10.1016/j.bodyim.2020.11.006

[71] K. A. Bailey , M. Dagenais , and K. L. Gammage , “Is a Picture Worth a Thousand Words? Using Photo‐Elicitation to Study Body Image in Middle‐To‐Older Age Women With and Without Multiple Sclerosis,” Qualitative Health Research 31, no. 8 (2021): 1542–1554. 34027715 10.1177/10497323211014830 PMC8371288

[72] C. Rice , S. Riley , A. LaMarre , and K. A. Bailey , “What a Body Can Do: Rethinking Body Functionality Through a Feminist Materialist Disability Lens,” Body Image 38 (2021): 95–105. 33839649 10.1016/j.bodyim.2021.03.014

[73] J. M. Alleva , T. L. Tylka , C. Martijn , M. I. Waldén , J. B. Webb , and N. Piran , “I'll Never Sacrifice My Well‐Being Again: The Journey From Negative to Positive Body Image Among Women Who Perceive Their Body to Deviate From Societal Norms,” Body Image 45 (2023): 153–171. 36934560 10.1016/j.bodyim.2023.03.001

[74] M. Tiggemann , “Considerations of Positive Body Image Across Various Social Identities and Special Populations,” Body Image 1, no. 14 (2015): 168–176. 10.1016/j.bodyim.2015.03.002 25865460

[75] J. M. Chevalier , Participatory Action Research: Theory and Methods for Engaged Inquiry, 2nd ed. (Routledge, 2019), 434p.

[76] K. A. Bailey and K. L. Gammage , “Applying Action Research in a Mixed Methods Positive Body Image Program Assessment With Older Adults and People With Physical Disability and Chronic Illness,” Journal of Mixed Methods Research 14, no. 2 (2020): 248–267.

[77] J. Brunet , J. Price , A. Baillot , E. Dann , and M. F. Vani , “Feasibility and Acceptability of Study Methods and Psychosocial Interventions for Body Image Among Women Diagnosed With Breast Cancer: A Systematic Review and Narrative Synthesis,” Psycho‐Oncology 33, no. 1 (2024): e6278. 38282235 10.1002/pon.6278

[78] V. White , J. Linardon , J. E. Stone , et al., “Online Psychological Interventions to Reduce Symptoms of Depression, Anxiety, and General Distress in Those With Chronic Health Conditions: A Systematic Review and Meta‐Analysis of Randomized Controlled Trials,” Psychological Medicine 52, no. 3 (2022): 548–573. 32674747 10.1017/S0033291720002251

[79] D. Brandenbarg , S. W. Maass , O. P. Geerse , et al., “A Systematic Review on the Prevalence of Symptoms of Depression, Anxiety and Distress in Long‐Term Cancer Survivors: Implications for Primary Care,” European Journal of Cancer 28, no. 3 (2019): e13086. 10.1111/ecc.13086 PMC9286037 31087398

